# Accuracy of Nanopore Sequencing as a Diagnostic Assay for Pulmonary Tuberculosis versus Smear, Culture and Xpert MTB/RIF: A Head-to-Head Comparison

**DOI:** 10.3390/tropicalmed8090441

**Published:** 2023-09-08

**Authors:** Juan Yang, Wei Ye, Chao Zhang, Wenhong Lin, Lin Mei, Shengsheng Liu, Jie Liu

**Affiliations:** 1 Department of Tuberculosis, Anhui Chest Hospital, Anhui Provincial Institute for Tuberculosis Prevention and Treatment, Hefei 230022, China; fanxingminiii@163.com (J.Y.); zhangchao210224@163.com (C.Z.); wenhonglin130@sina.com (W.L.); meilin@stu.ahmu.edu.cn (L.M.); 2 Department of Pathology, Anhui Chest Hospital, Anhui Provincial Institute for Tuberculosis Prevention and Treatment, Hefei 230022, China; ahsxkyyyw@163.com; 3 Department of Tuberculosis Control and Prevention, Anhui Chest Hospital, Anhui Provincial Institute for Tuberculosis Prevention and Treatment, Hefei 230022, China

**Keywords:** nanopore sequencing assay, tuberculosis, Xpert MTB/RIF, diagnosis

## Abstract

Early diagnosis of pulmonary tuberculosis (PTB) is pivotal for achieving effective tuberculosis (TB) control. This study aimed to assess the effectiveness of nanopore sequencing of sputum, bronchoalveolar lavage fluid (BALF), and pleural fluid samples for achieving early PTB diagnosis and provided head-to-head comparisons of nanopore sequencing results versus results obtained using smear, culture, and Xpert MTB/RIF assays. Patients admitted from October 2021 to April 2023 were screened for PTB using diagnostic imaging and electronic medical records. A total of 172 patients (129 PTB, 43 non-TB patients) were included in the final analysis after the exclusion of patients who did not meet the study’s inclusion criteria. PTB-positive rates were determined for each assay, and then, assay diagnostic efficacies were compared. The positive MTB-detection rates obtained using nanopore sequencing were 86.8% for all samples, 62.3% for BALF, and 84.6% for pleural fluid, all of which were significantly higher than the corresponding rates obtained using the other three assays. The overall sensitivity rates, specificity rates, and area under the curve (AUC) values obtained from smear testing were 5.4%, 95.3%, and 0.504, respectively, as compared to the respective results obtained via culture (18.6%, 100.0%, and 0.593), Xpert MTB/RIF (26.4%, 97.7%, and 0.620), and nanopore sequencing (85.3%, 95.4%, and 0.903). The diagnostic efficacy of nanopore sequencing surpassed the diagnostic efficacies of smear, culture, and Xpert MTB/RIF assays. Thus, nanopore sequencing holds promise as an alternative to Xpert MTB/RIF for early PTB detection, particularly for the testing of BALF and pleural fluid samples.

## 1. Introduction

Tuberculosis (TB) remains a serious threat to global health. According to a recent World Health Organization (WHO) report, 2021 witnessed an estimated 10.6 million new cases of TB and 1.6 million TB-related deaths [1]. TB is caused by Mycobacterium tuberculosis (MTB), an airborne pathogen that most commonly infects the lungs (pulmonary tuberculosis, PTB); however, MTB infections can occur in any organ (extrapulmonary tuberculosis, EPTB) [2]. Importantly, early detection of PTB cases and timely initiation of appropriate treatments are crucial measures for reducing TB transmission.

Smear microscopy is typically used for the initial TB diagnosis but has limited sensitivity and specificity in distinguishing between nontuberculous mycobacteria (NTM) and MTB, thus posing a diagnostic challenge [3]. Alternatively, MTB culture is currently regarded as the gold-standard TB diagnostic method, although this method is time-consuming and requires strict adherence to microbiological safety protocols [4,5]. Nonetheless, the rapid advancement of molecular technologies has led to the development of improved assays for achieving early and rapid diagnosis of infectious diseases, including TB [6,7]. Such tests target pathogen-specific molecular markers and genes associated with drug resistance [8]. One such assay, Xpert MTB/RIF (Cepheid Inc, Sunnyvale, CA, USA), is an automated, rapid, cartridge-based real-time nucleic acid amplification-based test that has been recommended by the WHO for use as an initial TB diagnostic assay for testing of patients with symptoms suggestive of TB [2,9,10]. Notably, the next-generation version of this assay, Ultra Xpert MTB/RIF (Cepheid Inc, Sunnyvale, CA, USA), provides greater sensitivity than Xpert MTB/RIF [2,11]. Regardless, the limited availability and affordability of these molecular assays have limited their widespread routine use in resource-limited settings [12]. In addition, low assay sensitivity associated with the testing of samples containing low bacterial loads is a serious concern [6]. Consequently, TB diagnostic assays with greater sensitivity and accuracy are urgently needed, particularly in low-income countries with high TB incidence rates.

In recent years, gene sequencing technology has played an increasingly important role in TB diagnosis with next-generation sequencing (NGS) technologies emerging as powerful tools for generating comprehensive, whole-genome sequence data at a single nucleotide level of resolution [13,14]. In fact, the use of NGS-based assays has resulted in improved diagnostic precision, treatment outcomes, and infectious disease monitoring. With regard to mycobacterial diseases, NGS has been successfully used to diagnose TB and numerous non-tuberculous mycobacterial infections, although high costs, labor-intensiveness, and sophisticated equipment requirements associated with NGS methods have impeded the widespread use of NGS in countries with high TB burdens. For example, a study on the budget impact of NGS for the diagnosis of TB drug resistance in Moldova showed that, for the phenotypic drug susceptibility testing scenario, the total cost is estimated to be US $362,000 over the 5-year study period, while the total cost of the NGS scenarios ranged from US $475,000 to US $1,486,000 [15]. To address these issues, nanopore sequencing, a portable third-generation sequencing technology developed by Oxford Nanopore, is an attractive NGS alternative with long-read sequence capability and high detection accuracy that only requires compact, low-cost hardware for implementation [13]. In recent years, nanopore sequencing has been increasingly used to identify pathogenic microbes [16,17], including those that cause non-infectious and infectious diseases and MTB with and without drug resistance; however, the use of this method for the testing of samples collected from patients with suspected early-stage mycobacterial infections has been limited [18]. Therefore, this study aimed to evaluate the effectiveness of nanopore sequencing as a diagnostic assay for detecting early PTB cases based on the testing of clinical specimens, such as sputum, lavage fluid, and pleural fluid. Furthermore, head-to-head comparisons of diagnostic performance were conducted between nanopore sequencing and acid-fast bacilli (AFB) smear, culture, and Xpert MTB/RIF assays.

## 2. Materials and Methods

### 2.1. Study Design and Population

**Figure 1:**
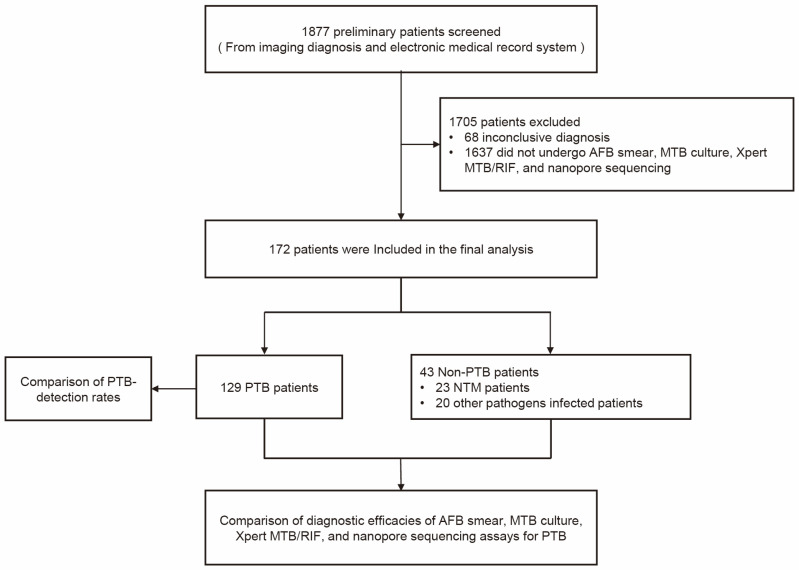
Study flowchart. AFB, acid-fast bacilli; MTB, *Mycobacterium tuberculosis*; PTB, pulmonary tuberculosis; NTM, nontuberculous mycobacteria.

This retrospective study was performed at Anhui Chest Hospital, Anhui Province, China. The study subjects included patients who were preliminarily diagnosed with PTB upon admission to the hospital from October 2021 to April 2023. The initial diagnoses that were established based on diagnostic imaging and electronic medical records were used to select patients for potential inclusion in the study. Routine testing of the patients with suspected PTB was conducted, and then, sputum, BALF, or pleural fluid samples were collected from the patients according to disease status. For example, BALF samples were collected via electronic bronchoscopy with alveolar lavage at the lesion site from patients who were unable to provide adequate amounts of sputa or from patients with smear-negative sputa (after all patients signed informed consent forms). Pleural fluid samples were obtained with thoracic puncture under B-type ultrasonic guidance from patients with pleural effusions who had provided informed consent. Throughout the testing phase, all patients received clinical follow-up care until a definitive diagnosis was established. The patients whose clinical specimens were not concurrently tested via AFB smear, MTB culture, Xpert MTB/RIF, and nanopore sequencing were excluded from the study, as were the patients lacking confirmed diagnoses. The workflow of the study is depicted in Figure 1.

The final clinical diagnosis for each patient with PTB was established in accordance with the criteria outlined in the standard Diagnosis of Pulmonary Tuberculosis Guidelines of China (WS288-2017). Within this study, the patients with bronchial TB and tuberculous pleurisy were classified as PTB cases. Clinically diagnosed PTB cases complied with one or more of the following criteria: (i) Patients who provided a respiratory tract sample or pleural fluid sample that yielded a positive culture for MTB; (ii) Patients with positive pathologic results indicative of PTB; (iii) Patients with typical clinical TB symptoms (such as subacute cough, hemoptysis, fever, night sweats, and/or weight loss); patients with positive results of immunological tests, such as the purified protein derivative (PPD) assay and/or gamma interferon (IFN-γ) release assay; chest radiograph findings suggestive of PTB; a positive clinical response to anti-TB treatment.

### 2.2. Specimen Collection

Early-morning sputum specimens were collected before the initiation of anti-infective therapy. BALF samples were collected using electronic bronchoscopy with alveolar lavage at the lesion site. Pleural fluid samples were obtained with thoracic puncture guided with B-type ultrasonography. Each specimen was divided into several parts for testing via AFB smear, MTB culture, Xpert MTB/RIF, and nanopore sequencing assays.

### 2.3. AFB Smear and MTB Culture Assays

AFB smears and MTB cultures were generated from clinical samples in accordance with standard practices [19]. For the smear testing, appropriate clinical specimens were digested, decontaminated, and centrifuged, and then, the NALC–NaOH method was used to prepare concentrated smears. The smears were stained with Auramine O (AO), a fluorescent stain, before conducting direct smear microscopy. The results were subsequently confirmed using Ziehl–Neelsen staining. For the MTB culture, the resuspended sediment was inoculated into liquid culture medium (MGIT, Becton Dickinson Diagnostics; Franklin Lakes, NJ, USA) for a 6-week incubation period.

### 2.4. Xpert MTB/RIF

For sample processing, a 2 mL volume of sputum, a 5 mL volume of centrifuged BALF, or a 5 mL volume of pleural fluid was thoroughly mixed with sample processing solution (containing sodium hydroxide and isopropanol) in a 2:1 volume ratio for 20 min with agitation. Next, the mixture was left to stand at room temperature for 15 min, and then, it was transferred to a reaction cartridge provided with the MTB/RIF detection kit (Cepheid, Sunnyvale, CA, USA). Thereafter, the cartridge was inserted into the test module of the GeneXpert instrument (Cepheid, Sunnyvale, CA, USA) for MTB detection. The Xpert MTB/RIF assay employs three specific primers and five unique molecular probes that target the 81 bp core region of the gene that encodes the mycobacterial RNA polymerase β subunit (rpoB) to thereby enable assay-based detection of MTB and DNA mutations that confer resistance to rifampicin. The GeneXpert instrument produced final MTB detection results within two hours.

### 2.5. Nanopore Sequencing

For sputum samples, a 400 μL volume of sputum sample was combined with an equal volume of NaOH solution, resulting in liquefaction of the sample. Next, the mixture was centrifuged (20,000× *g* for 10 min), and then, the supernatant was removed. For BALF and pleural fluid samples, 5 mL volumes of samples were centrifuged (20,000× *g* for 10 min), and then, the supernatants were discarded, and each pellet was resuspended in 200 μL of lysis solution. Using a previously reported method [20,21], genomic DNA was extracted from these specimens, and then, the DNA content was quantified using a Qubit 4.0 fluorometer (Life Technologies, Carlsbad, CA, USA). Prior to genomic DNA library preparation for nanopore sequencing, the DNA was further purified and then dissolved in TE buffer. Thereafter, the DNA concentrations were estimated, and the DNA was assessed for purity using a Nanodrop spectrophotometer (Thermo Fisher Scientific, Waltham, MA, USA) with blanks containing TE buffer read with each batch as a negative control. Each DNA sample that was tested via nanopore sequencing was required to have an OD 260/280 ratio of approximately 1.8 and an OD 260/230 ratio ranging from 2.0 to 2.2.

The PCR mix used for the nanopore sequencing assay was prepared according to the instructions included with the LongAmp Taq 2× Master Mix Kit (#M0287, NEB, Illumina, San Diego, CA, USA). The initial PCR cycling step (94 °C for 2 min) was followed by 30 three-step cycles (94 °C for 15 s, 60 °C for 15 s, 68 °C for 40 s) and a final hold at 4 °C. Gradient dilution was conducted to quantify the PCR products (within an approximate range of 100–200 fmol), and then, equimolar amounts of the PCR products were mixed, and a portion of each mixture was sent to ShengTing Bioinformatics Institute for sequencing. A Ligation Sequencing Kit (SQK-LSK109; ONT, Oxford, UK) and Native Barcoding Kit (EXPNBD104 and EXP-NBD114; ONT, Oxford, UK) were used to prepare multiplex PCR amplicons from the above-mentioned PCR samples. As per the Native Barcoding Kit protocol, end-prep followed by native barcode ligation to the amplicons were performed for approximately 3 h for each 100–200 fmol sample diluted in 65 μL of nuclease-free water. Adapter ligation and purification of the modified samples were then carried out using a NEB ligation kit and Agencourt AMPure XP beads (Beckman Coulter, Indianapolis, IN, USA), respectively, to generate a final adapter-ligated DNA library containing 50–100 fmol of DNA. DNA sequencing of the library was conducted using a GridION instrument (ONT) equipped with an R9.4 flow cell (ONT) with 800 effective pores. Following the sequencing run, the flow cell was cleaned using an ONT Flow Cell Wash Kit (EXP-WSH004) and stored at 4 °C. Data collection was conducted using the GridION platform (ONT) and MinKNOW v3.6.0 (ONT). DNA sequences that were shorter than 200 base pairs in length were filtered out. In order to align the sequences containing repetitive regions with the reference sequences, the repetitive regions were masked. Next, Minimap2 software was used to align the remaining sequences [22], remove the reads aligning with the human reference genome (GRCh38), and compare the remaining filtered reads to the MTB reference sequence (NC_000962.3) and Mycobacterium sequences (txid 1763) to detect MTB and nontuberculous mycobacteria (NTM), respectively, within 48 h.

### 2.6. Statistical Analysis

The statistical analysis was conducted using SPSS (IBM SPSS Software, version 25.0, SPSS Inc, Chicago, IL, USA) and MedCalc (MedCalc Software, version 19.5.6, Mariakerke, Belgium). Enumeration data were expressed as percentages. Paired data were compared using McNemar’s test. For each diagnostic parameter, assay sensitivity, specificity, positive predictive value, and negative predictive value were calculated concurrently. To evaluate the diagnostic efficacy of each assay, receiver operating characteristic (ROC) curves were generated and then used to determine the area under the curve (AUC) values and calculate the AUC value differences between the assays using the DeLong test. Statistical differences were considered significant at *p* < 0.05.

## 3. Results

### 3.1. General Clinical Data of Study Participants

A total of 1877 individuals were initially screened for PTB using diagnostic imaging and electronic medical records. Subsequently, 1705 individuals with inconclusive diagnoses were excluded from testing via AFB smear, MTB culture, Xpert MTB/RIF, and nanopore sequencing assays and, thus, were not included in the final analysis. The remaining 172 patients (aged 10 to 82 years) were included in the final analysis of which 129 were diagnosed with PTB and 43 with non-PTB disorders. Ultimately, 23 of the non-PTB patients were found to be infected with NTM, while the remaining 20 were found to harbor infections caused by other pathogens (Figure 1). Each patient provided one specimen, resulting in the collection from all patients of 8 sputum specimens, 151 BALF specimens, and 13 pleural fluid specimens. No patients were infected with HIV. General patient data are presented in Table 1.

### 3.2. PTB-Detection Rates of Smear, Culture, Xpert MTB/RIF and Nanopore Sequencing Assays

**Figure 2:**
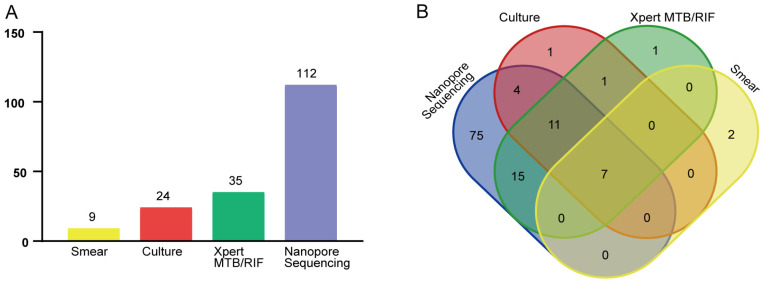
Positive PTB-detection rates obtained for smear, culture, Xpert MTB/RIF, and nanopore sequencing assays. (**A**) Number of samples yielding positive PTB-detection results for the four assays. (**B**) Venn diagram of MTB-positive results obtained for the four assays.

**Figure 3:**
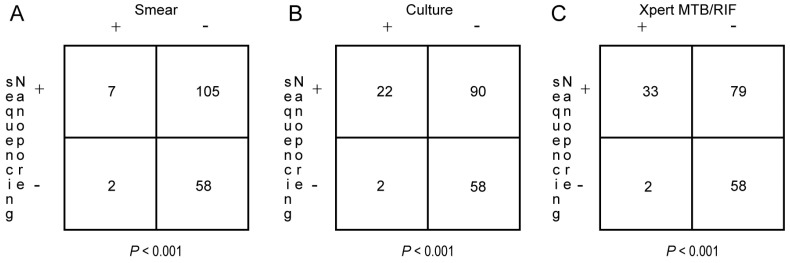
Pairwise comparisons between PTB-detection rates of nanopore sequencing and corresponding rates obtained using the other three methods (McNemar’s test). (**A**) Comparison of PTB-detection rates between nanopore sequencing and AFB smear assays. (**B**) Comparison of PTB-detection rates between nanopore sequencing and MTB culture assays. (**C**) Comparison of PTB-detection rates between nanopore sequencing and Xpert MTB/RIF.

Smear, culture, Xpert MTB/RIF, and nanopore sequencing assays yielded positive results for 9 (7.0%), 24 (18.6%), 35 (27.1%), and 112 (86.8%) specimens collected from PTB cases, respectively. The distribution and overlap of positive results obtained for each type of assay are shown in Figure 2. Significantly higher PTB-detection rates were obtained via nanopore sequencing as compared to the corresponding rates obtained for smear, culture, and Xpert MTB/RIF assays (McNemar’s test, all *p*-values were <0.001; Figure 3). Further analysis of nanopore sequencing performance in detecting MTB in sputum, BALF, and pleural fluid specimens produced results that are presented in Table 2. Notably, nanopore sequencing achieved a PTB-detection rate of 62.3% and 84.6% for BALF and pleural fluid specimens, respectively, which were significantly higher than the corresponding rates obtained using the other three detection methods (McNemar’s test, all *p*-values were < 0.05).

### 3.3. Comparison of Diagnostic Efficacies of Smear, Culture, Xpert MTB/RIF and Nanopore Sequencing Assays

**Figure 4:**
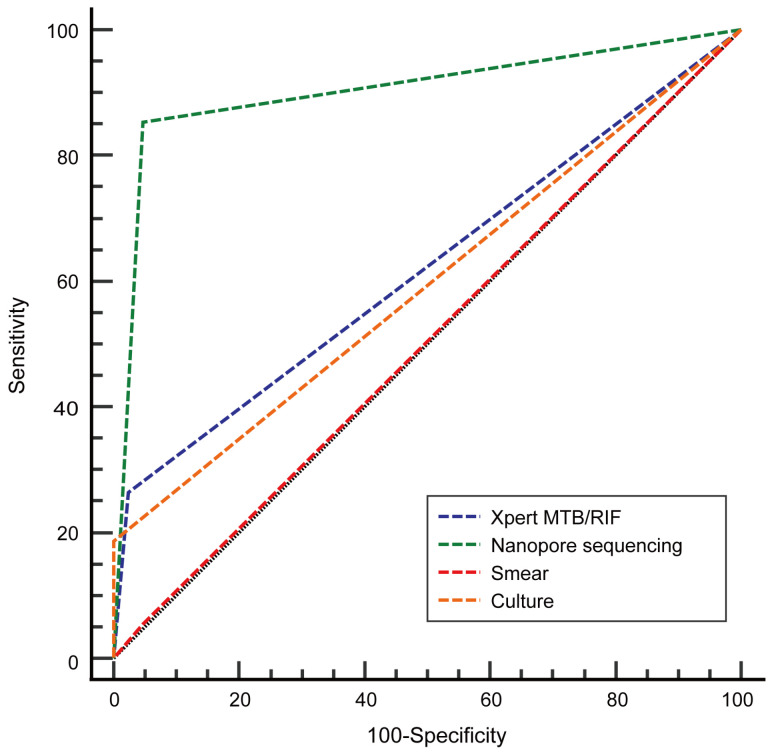
Receiver operating characteristic (ROC) curve-based PTB-detection results obtained using the four diagnostic assays. The area under the curve (AUC) value obtained for nanopore sequencing was 0.903, while the corresponding values obtained via Xpert MTB/RIF, culture, and smear were 0.620, 0.593, and 0.504, respectively.

The sensitivity rate, specificity rate, positive predictive value (PPV), and negative predictive value (NPV) obtained via AFB smear were 5.4%, 95.3%, 77.8%, and 25.2%, respectively, as compared to the respective values obtained via culture (18.6%, 100.0%, 100.0%, and 29.1%), Xpert MTB/RIF (26.4%, 97.7%, 97.1%, and 30.7%), and nanopore sequencing (85.3%, 95.4%, 98.2%, and 68.3%) with results shown in Table 3. The generation of ROC curves for further analysis (Figure 4) enabled comparisons of the ROC curve AUC values for nanopore sequencing to the corresponding values obtained for the other three tests (DeLong test). The AUC values obtained via nanopore sequencing were significantly higher than the AUC values obtained via smear (0.903 vs. 0.504, *p* < 0.001), culture (0.903 vs. 0.593, *p* < 0.001), and GeneXpert MTB/RIF (0.903 vs. 0.620, *p* < 0.001).

## 4. Discussion

PTB remains a major public health challenge with early and accurate PTB diagnosis playing a pivotal role in controlling the spread of the disease. Nevertheless, effective PTB control has been difficult to achieve due to atypical and insidious early PTB clinical manifestations [23]. Although radiologic findings can help clinicians diagnose PTB cases, they often fail to provide definitive confirmation of a PTB diagnosis. In instances where pathogenic evidence cannot be obtained, a diagnostic anti-TB treatment can be administered to patients in order to confirm or refute a PTB diagnosis, although many patients are reluctant to undergo such treatments due to the potential adverse effects of anti-TB drugs [24]. In recent years, molecular technological advances have enabled the development of laboratory assays for use in detecting MTB infections [2,7]. However, such methods developed to date have several disadvantages, as described above in the Introduction. Therefore, more rapid and accurate PTB diagnostic methods are needed, especially for patients who are unable to provide sputum.

Nanopore sequencing has garnered considerable attention due to its adaptability for use in real-time analysis, its ability to generate long sequencing reads with low error rates, and the current availability of data analysis software [18]. Nanopore sequencing assay kits were originally applied to genome sequencing when they were first placed on the market [25]. Since that time, advancements in sequencing chemistry and computational capacity have enabled the development of clinically applicable nanopore sequencing assays, including assays for detecting mycobacteria with and without drug resistance [18,26,27]. However, previously reported studies of nanopore sequencing assays with excellent pathogen detection capabilities have predominantly involved the testing of clinical culture-derived isolates or sputum specimens. In this study, we analyzed the clinical diagnostic value of a nanopore sequencing assay for the testing of BALF and pleural fluid specimens collected from patients with PTB. We then compared the MTB-detection efficacy of this assay to the corresponding efficacies of three etiological diagnostic methods commonly used in clinical practice: AFB smear, MTB culture, and Xpert MTB/RIF.

In this study, 172 patients were included in the final analysis of whom 129 were diagnosed with PTB and 43 with non-PTB infections. Of the 129 clinical samples obtained from the patients with PTB, 112 samples yielded MTB-positive detection results when tested via the nanopore sequencing assay, a higher MTB-detection rate than the corresponding rates obtained using the other three detection methods. In addition, nanopore sequencing was able to detect all 23 patients who were ultimately diagnosed with NTM infections. Taken together, these results indicate that the nanopore sequencing assay can be used to detect infections caused by *Mycobacterium* spp. and can distinguish MTB from NTM, consistent with the results of previous studies [28,29]. This result is of great importance since rapid discrimination between MTB and NTM is required to ensure that specific and effective medications are administered to patients in order to maximize the effectiveness of anti-mycobacterial treatments. Hence, use of a nanopore sequencing assay that can discriminate between MTB and NTM infections may improve the clinical management of patients with mycobacterial infections.

In clinical practice, sputum specimens are commonly used to obtain an etiological diagnosis of PTB. However, in instances when patients lack sputa or provide sputa that test negative for pathogens, collection of BALF or pleural effusion fluid is crucially important for diagnosing these patients. Thus, further analysis was conducted to assess the performance of the nanopore sequencing assay as compared to that of each of the other three assays when used to test different types of specimens. For pathogen detection in sputum samples, all four assays provided good pathogen-detection rates, although these results should be interpreted with caution since both the sample size and differences in MTB-detection performance between the assays were small. Nevertheless, nanopore sequencing-based testing of BALF specimens generated the highest MTB-detection rate (62.3%) followed by sputum tested via Xpert MTB/RIF (19.2%) and culture (11.3%). Importantly, the higher MTB-detection rate obtained for nanopore sequencing-based testing of BALF specimens suggests that this assay would be an effective test for diagnosing early-stage, smear-negative patients with PTB in clinical practice.

Exudative pleural effusions predominantly arise from malignant, tuberculous, and parapneumonic etiological origins with tuberculous pleural effusion reported to be particularly common in developing countries [30,31]. Unfortunately, unsuitably low MTB-detection rates are obtained via traditional TB diagnostic testing of tuberculous pleurisy patient pleural fluid samples, whereby only 10–35% of true-positive culture results and 20–81% of true-positive molecular test results actually demonstrate MTB detection in pleural fluid [31]. To our knowledge, few studies have explored the ability of nanopore sequencing to detect MTB in pleural fluid samples, prompting this study. Here, the results of nanopore sequencing, smear, culture, and Xpert MTB/RIF testing of 13 patients who were ultimately diagnosed with tuberculous pleurisy revealed that smear testing failed to detect MTB, culture-based testing only detected MTB infection in one patient, Xpert MTB/RIF only detected MTB in two patients, and nanopore sequencing detected MTB in 11 patients. Therefore, these results collectively suggest that nanopore sequencing is a highly sensitive method for detecting MTB DNA in pleural fluid and, thus, may be useful for achieving early diagnosis of patients with tuberculous pleural effusion. Despite the limitations of this study, which included a small sample size, the results we obtained are encouraging and highlight the great potential of nanopore sequencing to improve the diagnosis of tuberculous pleurisy in clinical practice, pending further confirmation of our results in larger studies.

A few reported studies have highlighted the diagnostic value of respiratory tract samples (sputum or BALF) collected from patients with suspected PTB when tested via nanopore sequencing. For example, Yu et al. found that the sensitivity rate, specificity rate, PPV, and NPV of the nanopore sequencing assay for diagnosing PTB cases were 94.8%, 97.9%, 99.1%, and 88.7%, respectively [29]. Similarly, the results obtained by Liu et al., who conducted nanopore sequencing detection of BALF specimens obtained from 55 suspected PTB cases, showed values for the abovementioned indicators of 75.86%, 80.77%, 81.48%, and 75.00%, respectively [28]. Taken together, these results collectively demonstrated that nanopore sequencing assays can provide greater accuracy and sensitivity than the corresponding standard pathogen detection assays. In view of the higher MTB-detection rate of the nanopore sequencing assay as compared to the rates obtained for smear, culture, and Xpert MTB/RIF assays, we further evaluated the diagnostic accuracy of the nanopore sequencing assay and then compared it head-to-head with the diagnostic accuracies of the other three assays. Our results revealed that all four methods provided ideal PTB diagnostic specificity but varied in diagnostic sensitivity. Furthermore, nanopore sequencing provided greater PTB diagnostic accuracy than that of the other three assays and had a sensitivity rate of 85.3% and a specificity rate of 95.4%. Moreover, both the ROC curve and the AUC values were employed to visually assess the predictive power of each of the four assays. The AUC value obtained using nanopore sequencing (0.903) was higher than the AUC values obtained for the other three MTB-detection methods, thus confirming that nanopore sequencing provided greater diagnostic accuracy than the other three methods, including Xpert MTB/RIF. In light of the reported good performance of Xpert MTB/RIF as a diagnostic test for PTB, the WHO recommended its use as an early PTB diagnostic test in 2014 [9]. Nevertheless, in this study, the Xpert MTB/RIF PTB diagnostic sensitivity rate and AUC value were only 26.4% and 0.620 and, thus, were unsatisfactory. Xpert MTB/RIF detects a specific *rpoB* sequence found in *Mycobacterium* spp. belonging to the MTB complex; this DNA sequence is present as a single copy per genome, thus limiting the sensitivities of the assays based on the detection of this genetic marker. By contrast, the nanopore sequencing assay targets the IS6110 insertion sequence, which is present as 10–12 copies per MTB genome. In addition, the poor sensitivity of Xpert MTB/RIF observed in this work may be partly due to the heterogeneity in bronchial lavage sampling during bronchoscopy between patients, especially since most of the samples tested here via Xpert MTB/RIF were BALF specimens. Therefore, the results of this study indicate that nanopore sequencing may effectively detect MTB in BALF or pleural fluid samples collected from patients with suspected PTB who did not produce sputum or whose sputum specimens tested negative for MTB and, thus, may be an attractive alternative to Xpert MTB/RIF for these patients.

We acknowledge several limitations of this study. First, this study is based on a relatively small sample size, especially for sputum and pleural fluid samples, which may have biased our results. Thus, a larger study incorporating a greater number of samples is required to validate our results. Second, patient-to-patient differences in applied physiological saline volumes used for BALF collection may have impacted our BALF MTB-detection results. Therefore, in subsequent studies, we plan to use the same volume of saline for bronchial lavage of all patients in order to minimize result bias due to the differences in bronchial lavage. Third, some patients may have previously received antibacterial drug treatments with anti-TB effects (e.g., fluoroquinolones) in primary medical institutions prior to enrollment in the current study, which may have affected our test results. To address this issue, a prospective study should be performed to determine whether this factor influenced the reliability of our results. Fourth, sequence depth that was too low, high host genome background noise, and low microbial pathogen load may have led to false-negative and false-positive nanopore sequencing results. Hence, in clinical practice, sequencing results should be strictly interpreted based on patient clinical manifestations in combination with imaging signatures and other conventional clinical microbiological test results. Fifth, it has been shown that stool Xpert MTB/RIF may be a useful rule-in test for children above and below 5 years of age under evaluation for PTB [32], which is of great significance for patients from whom good quality sputum samples are difficult to obtain. However, the diagnostic value of nanopore sequencing in non-respiratory samples for TB was not explored in this study, which may be our future research direction.

## 5. Conclusions

Nanopore sequencing applied to clinical samples (sputum, BALF, and pleural fluid) provides excellent PTB diagnostic accuracy that is significantly greater than that provided with AFB smear, MTB culture, and Xpert MTB/RIF assays. In real-world clinical practice, nanopore sequencing stands out as a promising alternative to Xpert MTB/RIF when used for early PTB detection, particularly for the testing of BALF and pleural fluid samples. Ultimately, nanopore sequencing is worthy of clinical promotion to improve the accuracy of early diagnosis of PTB.

## Figures and Tables

**Table 1:** General data of patients included in this study.

Characteristics	Total (*n* = 172)	PTB (*n* = 129)	Non-PTB (*n* = 43)
Gender (n, %)			
Male	103	77	26
Female	69	52	17
Age (n, %)			
<18 years	9	8	1
18–64 years	137	101	36
≥65 years	26	20	6
Smoking			
No	140	106	34
Yes	32	23	9
Drinking			
No	160	118	42
Yes	12	11	1
Comorbidity			
No	48	36	12
Yes	124	93	31
Diabetes	15	13	2
Hypertension	18	14	4
Bronchiectasis	7	5	2
Anemia	29	22	7
Hypoproteinemia	32	28	4
Abnormal liver function	11	10	1
Leukocytopenia	15	12	3
Other	39	27	12

PTB: pulmonary tuberculosis; Non-PTB: Other infectious diseases of the lungs other than tuberculosis.

**Table 2:** Positive detection of sputum, BALF, and pleural fluid specimens with the four methods.

Sample	Test	Num. Positive/Tested	Positive Rate (%)	*p* Value
Sputum	Smear	5/8	62.5	0.500 ^a^
	Culture	5/8	62.5	0.500 ^b^
	Xpert MTB/RIF	5/8	62.5	0.500 ^c^
	Nanopore sequencing	7/8	87.5	
BALF	Smear	4/151	2.6	<0.001 ^a^
	Culture	17/151	11.3	<0.001 ^b^
	Xpert MTB/RIF	29/151	19.2	<0.001 ^c^
	Nanopore sequencing	94/151	62.3	
Pleural fluid	Smear	0/13	0.0	0.001 ^a^
	Culture	2/13	15.4	0.004 ^b^
	Xpert MTB/RIF	1/13	7.7	0.002 ^c^
	Nanopore sequencing	11/13	84.6	

BALF: bronchoalveolar lavage fluid; ^a^, ^b^, and ^c^ represent Nanopore sequencing compared with smear, culture, and Xpert MTB/RIF, respectively (McNemar’s test).

**Table 3:** The diagnostic ability of the four tests for pulmonary tuberculosis.

Test	Sensitivity (%, 95% CI)	Specificity (%, 95% CI)	PPV (%, 95% CI)	NPV (%, 95% CI)	AUC (95% CI)
Smear	5.4 (2.2–10.9)	95.3 (84.2–99.4)	77.8 (43.0–94.2)	25.2 (23.7–26.6)	0.504 (0.427–0.581)
Culture	18.6 (12.3–26.4)	100.0 (91.8–100.0)	100.0 (/)	29.1 (27.4–30.8)	0.593 (0.516–0.667)
Xpert MTB/RIF	26.4 (19.0–4.8)	97.7 (87.7–99.9)	97.1 (82.7–99.6)	30.7 (28.3–33.1)	0.620 (0.543–0.693)
Nanopore sequencing	85.3 (78.0–90.9)	95.4 (84.2–99.4)	98.2 (93.4–99.5)	68.3 (58.6–76.7)	0.903 (0.849–0.943)

PPV: positive predictive value; NPV: negative predictive value; AUC: area under the curve.

## Data Availability

The datasets generated during and/or analyzed during the current study are not publicly available but are available from the corresponding author on reasonable request.
